# Supplementary material for: Perceived Stress, Well-Being, and Academic Performance of University Students During the First COVID-19 Lockdown: A Study of Portuguese, Spanish, and Brazilian Students
Source: Healthcare (Basel). 2025 Feb 10;13(4):371. doi: 10.3390/healthcare13040371 (PMC11855475; doi:10.3390/healthcare13040371)
Supplement: Supplementary file 1 [file healthcare-13-00371-s001.zip › healthcare-3439953-supplementary.pdf]

Table S1. Category system based on the Job Demands and Resources Model.

Categories	Subcategories	Examples of responses
Demands	**Demands related to work overload** - consisting of responses whose content indicated students' perception of a higher workload (hours, assignments, assessment) compared to the face-to-face class period.	"Overload of tests and assignments, as a consequence of the readaptation of teaching and assessment methods"<br>"Having a more intensive schedule"<br>"Many more assignments"
	**Demands related to experiencing emotional distress** - consisting of responses that focused on students' negative psychological experiences (e.g., anxiety, uncertainty, depression) during the lockdown period, at both personal and academic levels.	"Anxiety"<br>"Fear of the pandemic situation"<br>"Fear of not being able to succeed academically"
	**Demands related to assessment** - consisting of responses whose content reflected the difficulties experienced by the students in terms of assessment (assignments and tests).	"End-of-semester assignments"<br>"Increased assessment elements"<br>"Online tests"
	**Demands related to remote classes** – consisting of responses regarding the difficulties in adapting to remote classes directly experienced by the students or perceived by them in the adaptation of teachers to remote classes as an obstacle to learning)	"Confusing classes"<br>"More difficult to follow online classes"<br>"Technologically challenged teachers"
	**Demands related to isolation** - consisting of responses regarding the students' feelings of isolation due to the experience of lockdown and its impacts at both personal and academic levels.	"Isolation"<br>"Having to be at home"<br>"Inability to study in a group"
	**Demands related to the management of family, professional and academic interests**.- consisting of responses reporting difficulties experienced by the students in managing and balancing work, academic, and family life.	"Separating time to study, work, be with the family and household tasks"<br>"Balancing work and studies"<br>"Increased workload and little time to study"
	**Demands related to practice / research impairment -** consisting of students' responses regarding the suspension of practical classes or the postponement of research projects due to lockdown.	"Lack of practical classes"<br>"Having to inevitably postpone my research"<br>"Compromised data collection"
(Lack of) Resources	**Lack of resources related to institutional support -** consisting of all the responses reporting difficulties experienced by the students due to the lack of institutional support on the part of the university administration and/or support provided by the faculty.	"Lack of teachers' availability"<br>"Disorganization on the part of the university I was attending"<br>"Lack of information from the academic services"

	**Lack of technological resources** - consisting of students' responses regarding difficulties arising from the lack of technological resources (e.g., access to an individual computer; no internet or unstable internet).	"No internet"<br>"Poor internet"<br>"Only having access to a computer at night to be able to do the assignments "
	**Lack of space resources** - consisting of students' responses regarding the lack of an appropriate space to study or attend online classes	"Shared space; problem of not being able to study in a quiet, private place"<br>"Lack of own space to study"<br>"Lack of a quiet space and privacy"
	**Lack of financial resources** - consisting of responses reporting the students' lack of financial resources	"Lack of financial resources"<br>"Late tuition fees"<br>"Cash"
	**Lack of bibliographic resources** - consisting of students' responses concerning the lack of bibliographic resources, either due to their online unavailability, or to the need to use library services, to which access was limited or barred.	"Lack of materials<br>"Lack of resources-books that are only found in the library"<br>"Lack of access to the library"
